# Sinus Tachycardia Following Administration of Naloxone in a Dog During Anesthetic Recovery

**DOI:** 10.3390/ani16121803

**Published:** 2026-06-11

**Authors:** Toshitsugu Ishihara, Li-Jen Chang

**Affiliations:** Department of Small Animal Clinical Sciences, Virginia-Maryland College of Veterinary Medicine, Blacksburg, VA 24061, USA

**Keywords:** fentanyl, prolonged recovery, naloxone, reversal, arrhythmias, dog

## Abstract

A 7-year-old, 38.3 kg, male neutered Labrador retriever presented to the Teaching Hospital for a consultation of Comprehensive Oral Health Assessment and Treatment. Nine months prior to the consultation, the patient presented to the ER service in the teaching hospital due to acute right facial swelling. To further evaluate the swelling in the ER, the patient was sedated by IV administration of dexmedetomidine and fentanyl, and ventricular premature contractions (VPCs) were confirmed. The facial swelling subsided afterward, but it recurred. Therefore, the dog was placed under general anesthesia to evaluate the recurrent facial swelling and undergo a dental procedure. After 55 min of discontinuation of isoflurane, no swallowing reflex was observed, making extubation not possible. Naloxone (0.01 mg/kg) was administered IV to reverse the effects of fentanyl. Within one minute of administering naloxone, the patient was extubated. However, the HR surged to 240 bpm, and sinus tachycardia was observed. Ninety minutes after naloxone IV, the HR was 105 bpm with normal sinus rhythm, gradually approaching the pre-anesthesia HR level (110 bpm). Veterinarians should recognize that administration of naloxone could induce arrhythmias. Therefore, continuous monitoring of ECG, pulse, and blood pressure is imperative when administering naloxone.

## Simple Summary

Fentanyl is widely used as part of balanced anesthesia in dogs to provide perioperative analgesia and anesthetic-sparing effects. Occurrence of opioid associated dysphoria during recovery is a known side effect of fentanyl administration. Naloxone, a non-selective opioid receptors antagonist, reverses the adverse effects associated with the administration of opioid agonists. Administering a low dose of naloxone (0.01 mg/kg) intravenously (IV) has been reported to reduce opioid-induced dysphoria without affecting analgesia. This case report describes the clinical presentation, management, and outcome of a dog that developed untypical sudden sinus tachycardia following administration of naloxone after receiving fentanyl constant rate infusion (CRI). Prolonged recovery was observed following a 4-h continuous infusion of fentanyl and naloxone (0.01 mg/kg) was administered IV to reverse the effects of fentanyl. Although extubation became possible immediately after administration of naloxone, the heart rate (HR) surged from 80 beats per minute (bpm) to 240 bpm with sinus tachycardia. The sudden marked sinus tachycardia was likely caused by an increase in catecholamines resulting from rapid fentanyl antagonism, which was not typically observed. It is important to recognize that administration of naloxone could induce arrhythmias. Therefore, continuous monitoring of Electrocardiography (ECG), pulse, and blood pressure is essential when administering naloxone.

## 1. Introduction

Fentanyl is a synthetic opioid that is 100 times more potent than morphine, has a rapid onset of action and a short duration of action, and exhibits high selectivity for μ-opioid receptors and full agonist activity [1,2]. Fentanyl is widely used as part of balanced anesthesia in dogs to provide perioperative analgesia and anesthetic-sparing effects [3,4]. However, the occurrence of opioid associated respiratory depression, bradycardia and dysphoria during recovery is a known side effect of fentanyl administration in both humans and dogs [5,6]. Opioids significantly disrupt autonomic balance by suppressing the sympathetic nervous system, inducing a resting, high-vagal state, while withdrawal symptoms cause severe autonomic hypersensitivity [7,8]. Naloxone is a competitive μ-opioid receptor antagonist with low affinity for κ and δ receptors, and it reverses the adverse effects associated with the administration of opioid agonists [9,10]. It has been recommended by the RECOVER guidelines that opioids associated cardiopulmonary arrest should be treated with naloxone (0.04 mg/kg) IV or intraosseously to completely reverse the effects of opioids in dogs under resuscitation [11]. The effects of a higher dose of naloxone (0.04 mg/kg) on opioid-induced analgesia in dogs are unclear, whereas administering a lower dose of naloxone (0.01 mg/kg) IV has been reported to reduce opioid-induced dysphoria without affecting analgesia [12]. Administration of naloxone is generally associated with an increase in heart rate, cardiac output and arterial blood pressure in humans and in dogs [13,14].

This case report describes the clinical presentation, management, and outcome of a dog that developed sudden sinus tachycardia following administration of naloxone after receiving fentanyl CRI.

## 2. Case Presentation

A 7-year-old, 38.3 kg (body condition score 6 out of 9), male neutered Labrador retriever presented to the Veterinary Teaching Hospital (VTH) of Virginia-Maryland College of Veterinary Medicine for a consultation of Comprehensive Oral Health Assessment and Treatment (COHAT). Nine months prior to the COHAT consultation, the patient presented to the VTH ER service due to acute right facial swelling, where a 2–3 cm firm, non-movable swelling of the maxilla below the right orbit was identified. At that time, no other issues were detected based on the results of physical examination (PE), and blood examinations. To further evaluate the swelling in the ER, the patient was sedated by IV administration of dexmedetomidine (3 mcg/kg; Dexdomitor, Zoetis, Parsippany, NJ, USA) and fentanyl (3 mcg/kg; Fentanyl Citrate Injection, Hospira, Lake Forest, IL, USA), and a fine needle aspiration was performed on the lesion. A mixed bacterial flora was identified by cytology, with no evidence of neoplasia. During the procedure, VPCs were confirmed by a board-certified veterinary criticist. The VPCs resolved spontaneously without interventions. Amoxicillin-clavulanic acid and carprofen were prescribed, and the swelling resolved within a few days. However, similar facial swelling was noticed again three weeks before the dog presented to the VTH for a pre-COHAT consultation. Reoccurrence of swollen maxilla below the right orbit was confirmed. A slab fracture to the cranial portion of right second molar (208) and chipping on the tip of left second molar (108) were noticed by the oral examination. Dental radiographs, dental cleaning, and extractions were planned under general anesthesia to evaluate the recurrent facial swelling. Since VPCs were observed after sedation with dexmedetomidine and fentanyl nine months ago, a consultation and examination were conducted at the VTH cardiology service; however, no abnormalities were detected. Although an electrocardiogram (ECG) was performed, a Holter monitor test was not performed. Blood examination results, including complete blood count and chemistry panel, were unremarkable. On the day of the dental procedure, the dog had not received any medications. The dog was bright, alert and responsive on the day of anesthesia with HR of 110 bpm, respiratory rate (RR) of 20 breaths per minute and a rectal temperature (T) of 38.9 °C. Thoracic auscultation revealed normal lung sounds with no murmurs or arrhythmias, and capillary refill time was less than two seconds. The dog was classified as an American Society of Anesthesiologists physical status II.

**Figure 1:**
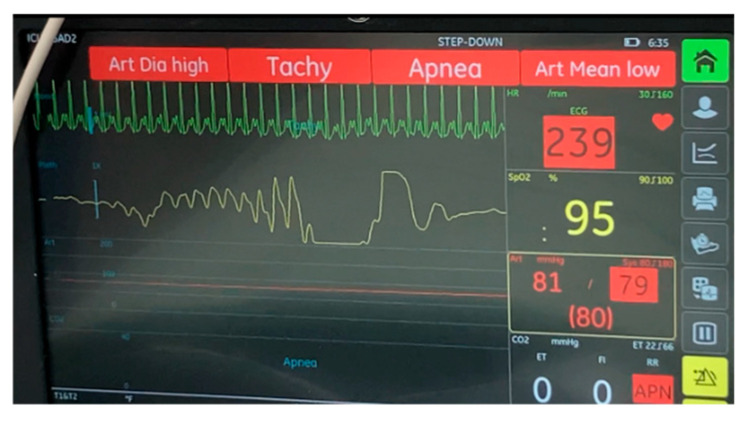
The HR reached a maximum of 240 bpm, and sinus tachycardia was observed following intravenous administration of naloxone (0.01 mg/kg). The MAP was 80–82 mmHg, and SpO_2_ was 95–97% under room-temperature air. This figure captured the moment when HR was 239 bpm, MAP was 80 mmHg and SpO_2_ was 95%. The decreased amplitude of the arterial blood pressure waveform is thought to be due to tachycardia, which shortens the diastolic phase, reduces stroke volume, and lowers systolic blood pressure. The pulse was detected on palpation.

**Figure 2:**
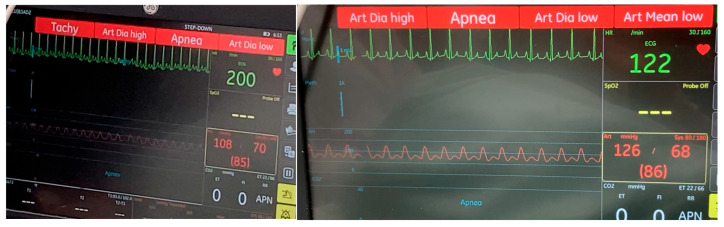
Twenty minutes after intravenous administration of naloxone, the HR was 200 bpm, and the MAP was 85 mmHg without treatment; 60 min after intravenous naloxone administration, the HR was 122 bpm, and the MAP was 86 mmHg.

The dog was premedicated intramuscularly (IM) in the semimembranosus muscle with acepromazine (0.02 mg/kg; Acepromazine Maleate Injection, VetOne, Boise, ID, USA) and hydromorphone (0.1 mg/kg; Hydromorphone Hydrochloride Injection USP; TEVA Pharmaceutical Inc., Parsippany, NJ, USA), and two 18-gauge intravenous catheters (Surflo I.V. Catheter, Terumo Medical, Somerset, NJ, USA) were placed into both cephalic veins after moderate sedation. An ECG was performed before and after the premedication, but no abnormalities were detected. The dog received 100% oxygen (4 L/min) through a mask for 3 min before induction of general anesthesia with midazolam (0.2 mg/kg; Midazolam Injection USP; Almaject Inc., Morristown, NJ, USA) IV and propofol (5.3 mg/kg; PropoFlo™-28, Zoetis Inc., Parsippany, NJ, USA) IV titrated to effect. After tracheal intubation, general anesthesia was maintained with a rebreathing circuit using isoflurane (Fluriso, VetOne, ID, USA) in 100% oxygen at a rate of 1–4 L/min. Maropitant citrate (1 mg/kg; Cerenia, Parsippany, NJ, USA) was administered subcutaneously (SC) after induction. A multiparameter monitor (Dash 4000; GE Healthcare, Chicago, IL, USA) was used to monitor ECG in lead II, peripheral oxygen saturation of hemoglobin (SpO_2_), RR, T, fraction of end-tidal isoflurane (ETIso) and carbon dioxide partial pressure (ETCO_2_), an invasive systolic, diastolic and mean arterial blood pressure (MAP) at 5-min intervals during anesthesia. Invasive blood pressure values were obtained through a 22-gauge catheter in the left radial artery. Intravenous fluid therapy was initiated with Lactated Ringer’s solution (LRS) (Vetivex Veterinary Lactated Ringer’s Injection, Dechra Veterinary Products, Overland Park, KS, USA) at the rate of 5 mL/kg/h (hour). The dog was provided thermal support using a forced air warming system (3M^TM^ Bair Hugger^TM^, Arizant Healthcare Inc., Eden Prairie, MN, USA) and an external heat pad (HotDog^TM^ Patient Warming System, Augustine Temperature Management LLC, Eden Prairie, MN, USA). Ten minutes after induction, HR was 75 bpm with normal sinus rhythm, RR was 20 breaths per minute, and MAP was 73 mmHg. Bilateral maxillary nerve blocks were performed using bupivacaine (0.4 mg/kg; Bupivacaine Hydrochloride Injection, Hospira Inc., Lake Forest, IL, USA). Fentanyl CRI (5 mcg/kg/h) and lidocaine (1 mg/kg; Lidocaine HCl 2% Injection, VetOne, ID, USA) were administered IV, followed by lidocaine CRI (1.5 mg/kg/h). The dog was transferred to a dental treatment room 35 min after induction. Mechanical ventilation (MV) (Ohmeda7900, Datex-Ohmeda, GE Healthcare, Chicago, IL, USA) was initiated due to hypercapnia (ETCO_2_ of 55 mmHg) with a setting of peak inspiratory pressure (PIP) of 10 cmH_2_O and RR of 10–12 breaths per minute. 15 min after the MV was set, the ETCO_2_ remained at 44 mmHg. During the procedure, hypotension (MAP 59 mmHg) was observed. The HR was 75 bpm, and the depth of anesthesia was assessed as Stage III (Stage of surgical anesthesia). As no obvious pulse pressure variation (PPV) and pleth variability index (PVI) were detected under MV, an intravenous fluid bolus was not administered to treat hypotension. Instead, norepinephrine CRI (0.1 mcg/kg/h; Norepinephrine Bitartrate Injection, Amneal Pharmaceuticals, Inc., Bridgewater, NJ, USA) was initiated. VPCs were noticed within a few minutes after norepinephrine CRI. The HR was 81 bpm at this moment. Norepinephrine CRI was discontinued because of no improvement in blood pressure and the VPCs. To have a further isoflurane-sparing effect to improve hypotension, fentanyl CRI was increased to 10 mcg/kg/h without a loading dose of fentanyl. Since the procedure was within the range covered by maxillary nerve blocks at that time, and a sudden drop in blood pressure caused by a rapid decrease in heart rate was anticipated due to the loading dose, the loading dose was not administered, although this is not standard practice. In addition, given that the HR was 75 bpm and that a decrease in HR was anticipated with the increased dose of fentanyl, glycopyrrolate (0.005 mg/kg; Glycopyrrolate Injection, Fresenius Kabi, Lake Zurich, IL, USA) was also administered IV. Fifteen minutes after administration of glycopyrrolate, HR was 120 bpm and MAP was 90 mmHg. Glycopyrrolate was administered three times throughout the procedure to maintain blood pressure. Fentanyl CRI and lidocaine CRI were discontinued 20 min prior to stopping isoflurane. Spontaneous breathing was confirmed 10 min after discontinuation of isoflurane. The duration of anesthesia was 5 h and 21 min. During the procedure, the ETIso was maintained between 1.2% and 1.5%, the T was maintained between 36.7 °C and 38.9 °C, the HR was between 66 and 122 bpm, the MAP was maintained between 59 mmHg and 90 mmHg, the SpO_2_ was between 95% and 100%, and the ETCO_2_ was between 44 mmHg and 55 mmHg. The total volume of LRS administered was 890 mL. The duration of the fentanyl CRI was 4 h. The dog was positioned in sternal recumbency for recovery. After 55 min of discontinuation of isoflurane, the dog remained in sternal position with eyeballs rotating ventromedially and no swallowing reflex observed, making extubation not possible. Since the prolonged recovery was thought to be a result of a 4-h fentanyl infusion, naloxone (0.01 mg/kg; Naloxone Hydrochloride Injection USP, Hospira Inc., Lake Forest, IL, USA) was administered IV to reverse the effects of fentanyl. Before administration of naloxone, T was 36.8 °C, HR was 80 bpm, RR was 12 breaths per minute, ETCO_2_ was 35 mmHg, and MAP was 90 mmHg. Within one minute of administering naloxone, the dog moved its head and swallowed, allowing us to extubate the endotracheal tube. After extubation, the dog maintained in sternal position without signs of agitation and breathed steadily. The dog was conscious and appeared to be aware of its surroundings. However, the patient fell asleep immediately when stimulation was absent. The HR was 240 bpm, and sinus tachycardia was observed after administration of naloxone (Figure 1). MAP was 80–82 mmHg, and SpO_2_ was 95–97% with room air. Palpation around the dental extraction area elicited no response in the dog. 5 mL/kg of LRS was administered over 5 min for fluid challenge, producing no changes in HR or MAP. Packed cell volume (PCV) was checked. And, arterial blood gas analysis and blood chemistry testing were performed. The results of arterial blood gas showed pH of 7.39, oxygen tension of arterial blood (PaO_2_) was 89 mmHg and arterial partial pressure of carbon dioxide (PaCO_2_) was 25 mmHg. The PCV and blood chemistry results were unremarkable. Based on the patient’s clinical symptoms, we suspected that the presence of sinus tachycardia was a result of acute opioid reversal by naloxone. Since, except for the tachycardia, the dog’s overall condition was stable and the relatively short duration of action of naloxone, we decided to closely monitor the dog without further interventions. Twenty minutes after naloxone, the HR dropped to 200 bpm and the MAP was 85 mmHg (Figure 2). Thirty minutes after naloxone, the HR was 186 bpm and the MAP was 84 mmHg. Following this, sixty minutes after naloxone IV, the HR was 122 bpm and the MAP was 86 mmHg (Figure 2), and ninety minutes after naloxone IV, the HR was 105 bpm with normal sinus rhythm and the MAP was 85 mmHg, gradually approaching the pre-anesthesia HR level (110 bpm). Carprofen (4.4 mg/kg) was administered SC after normal sinus rhythm and normal MAP were confirmed. The dog was able to walk on its own and was discharged without further complications. The dog’s health status was followed up and it remained clinically normal without further complications being reported.

## 3. Discussion

In this case, prolonged recovery was observed following a 4-h continuous infusion of fentanyl and naloxone (0.01 mg/kg) was administered IV to reverse the effects of fentanyl. We assume that the prolong recovery in this case is a result of fentanyl infusion because extubation became possible immediately after administration of naloxone. However, the HR surged from 80 bpm to 240 bpm with sinus tachycardia was noticed following naloxone IV. Sinus tachycardia is a sinus rhythm characterized by a faster-than-normal heart rate which is about heart rate higher than 160 bpm in large breed dogs [15]. Sinus tachycardia can be a result of pain, light anesthetic plane, anxiety, administering anticholinergics or sympathomimetics, such as dopamine or norepinephrine, thyroid over-supplementation, hyperthyroidism, fever, shock, chronic heart failure, or the early stages of hypoxia [16]. In this case, since the patient did not appear agitated, showed no signs of pain, had a normal body temperature and blood pressure, did not respond to the fluid challenge, and had normal blood test results, it was suspected that the sinus tachycardia was caused by naloxone. Ninety minutes after administration of naloxone, the HR had returned to its pre-anesthesia level. This is consistent with the pharmacokinetic profile of naloxone that the elimination half-life of IV administered naloxone is approximately one hour and its duration of action ranges from 45 to 180 min [17]. Naloxone associated cardiovascular adverse events, including severe hypertension, cardiac arrhythmias, and pulmonary edema have been sporadically reported in humans [18,19]. Ventricular tachycardia (VT) and ventricular fibrillation (VF) are potential complications caused by the administration of naloxone, although their occurrence is rare [20]. It has been recommended that when administering naloxone to specific patients, such as patients with a history of opioid abuse or patients receiving iatrogenic high opiate doses, a low dose be used, ECG monitoring be performed, and be ready for defibrillation [20]. Ventricular tachycardia or VF may be induced when the protective mechanism of opioids against excessive sympathetic activation—caused by drug abuse, heart disease, or hypoxia—is blocked by naloxone administration [20]. Furthermore, in humans, suppression of cardiopulmonary function is a leading cause of death from opioid overdose; in cases of overdose involving fentanyl or a combination of fentanyl and other drugs, higher doses and/or more frequent administration of naloxone are required. However, increasing the dose of naloxone also increases the risk of adverse cardiopulmonary effects, such as tachycardia and tachypnea [21]. In a study involving dogs, heart rate increased by 45% five minutes after administering naloxone IV to dogs that had received fentanyl and droperidol. However, no cardiac arrhythmias, including sinus tachycardia, were observed [22]. In another study, all dogs were administered an overdose of transdermal fentanyl solution (13.0 mg/kg). The HR decreased from 101 bpm before administration of the transdermal fentanyl solution to 64.2 bpm after administration. Subsequently, a 40 mcg/kg intramuscular dose of naloxone increased the HR to 94.4 bpm, and a 160 mcg/kg IM dose increased it to 104 bpm. In this study, HR was significantly higher in the group receiving a higher dose of naloxone than in the group receiving an injection of 40 mcg/kg of naloxone, but tachycardia was not observed [23]. Furthermore, in a study of dogs under enflurane-nitrous oxide anesthesia, in which approximately 0.019 mg/kg of naloxone was administered IV 50 min after a total fentanyl dose of 50 mcg/kg, the HR returned to the control level observed before fentanyl administration. Plasma norepinephrine and epinephrine concentrations reached 164% and 158%, respectively, of pre-fentanyl administration levels [24]. In another study using dogs, changes in hemodynamics following nalbuphine administration were not accompanied by an increase in plasma catecholamine concentrations. However, following subsequent administration of naloxone, plasma concentrations of both epinephrine and norepinephrine increased significantly [13]. Nevertheless, the increase in plasma catecholamine concentrations is not caused solely by the administration of naloxone [25]. In addition, while one study has shown that fentanyl diminishes sympathetic outflow in dogs, the institution of hypercapnia did elicit some adrenergic response even in these narcotized, well-anaesthetized animals. Furthermore, a bolus injection of 20 mcg/kg of naloxone caused a further increase in plasma catecholamine concentrations, as well as significant increases in heart rate and blood pressure [26]. To the author’s knowledge, however, there are no reports of arrhythmias such as sinus tachycardia or VT occurring in dogs following the administration of naloxone in clinical settings.

It is known that in humans, patients receiving high doses of iatrogenic opioids are at increased risk of developing arrhythmias following naloxone administration [20]. Therefore, in our canine case, naloxone administration after 4 h of fentanyl CRI may have caused a rapid rise in plasma catecholamine concentrations, leading to sinus tachycardia. However, the context-sensitive half-time (CSHT) of fentanyl in dogs is significantly shorter than in humans [27]. In humans, the CSHT of fentanyl is significantly prolonged 3 h after administration, which may contribute to cumulative effects [28]. In dogs, however, it has been suggested that the long-term infusion of fentanyl causes less delay in recovery after general anesthesia in dogs, even at the higher dose rates indicated for intraoperative use due to its shorter CSHT and less cumulative effects [27,29,30,31]. Therefore, in our case, it is expected that there was little accumulation of fentanyl in the body even after a 4-h CRI, and it is further expected that the accumulation of fentanyl was even lower when naloxone was administered 75 min after the discontinuation of the fentanyl CRI. However, the presence of marked sinus tachycardia following naloxone administered suggests that fentanyl remains accumulating, which could also explain the prolonged recovery from general anesthesia. While common causes of prolonged recovery include hypothermia, hypotension, hemorrhage, hepatic incompetence, renal dysfunction, hypoglycemia, neurological disorders, hypocalcemia, and drug overdose [32], none of these conditions are clearly applicable to this dog. Factors contribute to drugs greater accumulation than anticipated plasma concentrations include the function of canine polymorphism of the CYP2B11 enzyme system and CYP1A2 [33,34,35], and the adenosine triphosphate-binding cassette subfamily B, member 1 (ABCB1) gene mutation [36]. It is unclear whether these genetic factors were involved in this case.

VPCs were observed in this case following the previous administration of dexmedetomidine. Furthermore, VPCs were observed during the dental procedure when norepinephrine was administered. Norepinephrine is a potent vasopressor, its β1-adrenergic receptor-stimulating effects might cause arrhythmias, including VPCs [37]. Although a cardiology consultation revealed no obvious cardiac abnormalities in this patient, the patient’s heart might be predisposed to an abnormal response even to clinically relevant doses of medications. Therefore, it is possible that the abnormal sinus tachycardia observed is a response to the rapid rise in plasma catecholamine concentrations following administration of naloxone. Glycopyrrolate (0.005 mg/kg) IV was administered three times throughout the procedure to maintain blood pressure. The duration of action of glycopyrrolate is 1 h [38,39]. Considering the time elapsed since the administration and the total dose of 0.015 mg/kg, which is not particularly high, it is likely that the drug’s effects had already worn off by the time the sinus tachycardia occurred. However, it cannot be excluded that the rapid increase in heart rate was caused by the action of glycopyrrolate, possibly due to the antagonism of fentanyl by naloxone. Furthermore, acepromazine, which was administered for premedication, is known to have antiarrhythmic effects [40], and while it was administered for that purpose, it cannot be excluded that its vasodilatory effect, causing hypotension [40,41], contributed to the sinus tachycardia. It is also possible that the sinus tachycardia was caused not only by naloxone but by a combination of the factors mentioned above.

## 4. Conclusions

This case report describes the clinical signs, management, and outcome of the dog that developed sinus tachycardia following the administration of naloxone after receiving a 4-h fentanyl CRI. Although the sudden sinus tachycardia was likely caused by an increase in catecholamines resulting from rapid fentanyl antagonism, definitive causes for this marked sinus tachycardia, which is not typically observed, could not be identified in this dog. However, it is important to recognize that administration of naloxone could induce arrhythmias. Therefore, continuous monitoring of ECG, pulse, and blood pressure is essential when administering naloxone. Starting with a lower dose may also be preferable. Since the dog was administered numerous medications other than naloxone during the dental procedure, other causes, such as interactions with those medications, cannot be excluded.

## Data Availability

The datasets used and/or analyzed during the current study are available from the corresponding author upon reasonable request.

## Abbreviations

The following abbreviations are used in this manuscript:

IV	Intravenously
CRI	Constant rate infusion
HR	Heart rate
bpm	Beats per minute
ECG	Electrocardiography
VPCs	Ventricular premature contractions
VTH	Veterinary teaching hospital
COHAT	Comprehensive oral health assessment and treatment
PE	Physical examination
RR	Respiratory rate
T	Rectal temperature
IM	Intramuscularly
SQ	Subcutaneously
SpO_2_	Peripheral oxygen saturation of hemoglobin
ETIso	Fraction of end-tidal isoflurane
ETCO_2_	Carbon dioxide partial pressure
MAP	Mean arterial blood pressure
LRS	Lactated Ringer’s solution
h	hour
PPV	Pulse pressure variation and
PVI	Pleth variability index
PCV	Packed cell volume
PaO_2_	Oxygen tension of arterial blood
PaCO_2_	Arterial partial pressure of carbon dioxide
VT	Ventricular tachycardia
VF	Ventricular fibrillation
CSHT	Context-sensitive half-time
ABCB1	Adenosine triphosphate-binding cassette subfamily B, member 1
